# Feline Coronavirus 3c Protein: A Candidate for a Virulence Marker?

**DOI:** 10.1155/2016/8560691

**Published:** 2016-05-03

**Authors:** A. S. Hora, P. O. Tonietti, S. A. Taniwaki, K. M. Asano, P. Maiorka, L. J. Richtzenhain, P. E. Brandão

**Affiliations:** ^1^Department of Preventive Veterinary Medicine and Animal Health, School of Veterinary Medicine, University of São Paulo, Avenida Professor Dr. Orlando Marques de Paiva 87, Cidade Universitária, 05508-270 São Paulo, SP, Brazil; ^2^Coronavirus Research Group, School of Veterinary Medicine, University of São Paulo, Avenida Professor Dr. Orlando Marques de Paiva 87, Cidade Universitária, 05508-270 São Paulo, SP, Brazil; ^3^Department of Pathology, School of Veterinary Medicine, University of São Paulo, Avenida Professor Dr. Orlando Marques de Paiva 87, Cidade Universitária, 05508-270 São Paulo, SP, Brazil

## Abstract

Feline infectious peritonitis virus (FIPV) is highly virulent and responsible for the highly fatal disease feline infectious peritonitis (FIP), whereas feline enteric coronavirus (FECV) is widespread among the feline population and typically causes asymptomatic infections. Some candidates for genetic markers capable of differentiating these two pathotypes of a unique virus (feline coronavirus) have been proposed by several studies. In the present survey, in order to search for markers that can differentiate FECV and FIPV, several clones of the 3a–c, E, and M genes were sequenced from samples obtained from cats with or without FIP. All genes showed genetic diversity and suggested the presence of FCoV mutant spectrum capable of producing a virulent pathotype in an individual-specific way. In addition, all the feline coronavirus FIPV strains demonstrated a truncated 3c protein, and the 3c gene was the only observed pathotypic marker for FCoVs, showing that 3c gene is a candidate marker for the distinction between the two pathotypes when the mutant spectrum is taken into account.

## 1. Introduction

Feline coronavirus (FCoV) (*Nidovirales; Coronaviridae; Coronavirinae; Alphacoronavirus 1*) is a widespread pathogen among the domestic cat population. FCoV occurs as two pathotypes, feline enteric coronavirus (FECV) and feline infectious peritonitis virus (FIPV), both of which with strains of serotypes I and II [1].

Phenotypically, the main difference between the pathotypes is that FIPV is able to infect monocytes and macrophages, causing systemic infection and fatal disease, while FECV is limited to replication in the mature intestinal epithelium, which mainly results in asymptomatic infection. However, FECV may undergo a short systemic viremia phase that also involves monocytes [2, 3].

The FCoV genome (single-stranded positive-sense linear RNA, 29 kb) encodes proteins directly involved in viral replication (ORF1), the structural spike protein (S), envelope protein (E), membrane protein (M), and nucleocapsid protein (N), as well as the accessory proteins 3a–c and 7a-b [4].

There is a major controversy regarding the existence of genetic markers to differentiate between FECV and FIPV pathotypes [5, 6]. Numerous markers that correlate with the manifestation of FIP have been proposed for S [7–12], 3a–c [8, 13–16], 7a-b [16–18], and M [5] genes.

The most studied gene is 3c [7, 9, 13–15, 19, 20], which is present in the truncated form in the majority of all strains obtained from animals with FIP. However, previous studies on 3c genes focused only on the dominant sequence and not on the mutant spectrum or the clonal diversity and the information obtained from a dominant sequence may not be suitable to detect mutations [10], with the exception of a study [7] in which clones were used to assess the presence of alterations in the gene 3c in ascites and/or faeces from FIP and healthy cats, and again 3c truncated protein was not observed in 100% of FIPV samples.

Considering (i) the current discussion on molecular markers capable of distinguishing the pathotypes of FCoV and (ii) the lack of knowledge of the diversity of such markers in the mutant spectrum of RNA, the present study was designed to investigate the clonal diversity of the 3a–c, E, and M genes in FCoV strains present in tissue and/or faecal samples from cats with and without FIP.

## 2. Materials and Methods

### 2.1. Animals and Samples

From 2010 to 2013, tissue (eye, cerebrum, cerebellum, lung, heart muscle, thoracic lymph node, thymus, liver, spleen, stomach, mesenteric lymph node, peripancreatic lymph node, kidneys, large and small intestines, and urinary bladder), abdominal, thoracic, and pericardial effusions, aqueous humor, and faecal samples were collected during the necropsies of 6 cats with clinical manifestations and histopathological changes consistent with FIP, such as localized inflammation with macrophages, neutrophils, lymphocytes, and plasma cells. Vascular lesions were found surrounded by a proliferation of inflammatory cells in heart, lung, omentum, kidneys, cerebrum, and cerebellum. Focal accumulations of inflammatory cells and necrotic-proliferative lesions were observed in granulomatous lesions. Intestinal samples were collected including mucosa, submucosa, muscular layer, and serosa. Only 2 animals (USP6, and USP7) showed signs of dry-form FIP (neurological changes), while the remaining 4 (USP8, USP16, USP17, and USP18) showed an accumulation of effusion in body cavities (effusive FIP).

All FIP-positive cats were sampled from different geographic locations, with the exception of 2 cats (USP17 and USP18) that lived in the same house. USP17 was euthanized 1 month prior to USP18 due to FIP related reasons.

In parallel, faecal samples were also obtained from 2 cats (USP2, and USP14) without clinical signs of FIP and from two different locations not related to the locations from the FIP+ cats.

This study has been approved by Commission on Ethics on Animal Use from School of Veterinary Medicine, University of São Paulo (CEUA N. 2055211113).

### 2.2. Messenger RNA Detection for the M Gene of FCoVs

All tissues were complete homogenized after tissue maceration and before RNA extraction. Total RNA extraction and RT-PCR to detect mRNA of the M gene of FCoVs were performed in all samples as previously described [21] as a screening test for FCoV replication.

### 2.3. Amplification of the 3a–c, E, and M Genes of FCoVs

All samples from 6FIP+ and 2FIP− cats were submitted to amplification of the 3a–c, E, and M genes of FCoVs, even if the mRNA detection was negative. To amplify all these five genes of FCoVs in only one amplicon, a single pair of previously described primers [14] was used (nucleotides 24,380 to 26,996 corresponding to the FCoV-WSU 79-1146; genome GenBank DQ010921).

cDNA synthesis was performed with 6 *μ*L total RNA extracted with TRIzol*™* Reagent (Life Technologies Corporation, Carlsbad, CA, USA) and ThermoScript*™* (Life Technologies Corporation, Carlsbad, CA, USA) using Random Primers*™* (Life Technologies Corporation, Carlsbad, CA, USA) according to the manufacturer's instructions.

For PCR, 5 *μ*L of cDNA was added to 1x PCR buffer (Life Technologies Corporation, Carlsbad, CA, USA), 2 mM MgSO_4_, 0.2 mM of each dNTP, 0.25 *μ*M of each primer, 1.5 U Platinum*™* Taq DNA Polymerase High Fidelity (Life Technologies Corporation, Carlsbad, CA, USA), and DEPC water for a final reaction volume of 50 *μ*L. The cycling conditions were as follows: 94°C/2 min; 10 cycles of 94°C/15 s, 50°C/15 s with an increase of 0.5°C per cycle, and 68°C/4 min; 10 cycles of 94°C/15 s, 55°C/15 s, and 68°C/4 min; 25 additional cycles of 94°C/15 s, 55°C/15 s, and 68°C/4 min with an increase of 10 s per cycle; and a final extension of 72°C/10 min.

Specific bands were excised from the gels and purified using the Gene Jet*™* Gel Extraction kit (Thermo Fisher Scientific Inc., Vilnius, Lithuania) according to the manufacturer's instructions.

### 2.4. Molecular Cloning

The purified amplicons (2,617 bp containing 3abc-E-M) were cloned using pGEM*™*-T Easy Vector Systems (Promega Madison, WI, USA) and* E. coli* JM109 competent cells according to the manufacturer's recommendations. After the transformation protocol, transformation culture was plated onto duplicate LB/ampicillin/IPTG/X-Gal plates and the plates were incubated overnight (16 hours) at 37°C. Then, the transformed colonies were transferred to LB liquid medium and were incubated overnight (16 hours) at 37°C and 180 rpm. Recombinant plasmid extraction was performed with the Illustra*™* plasmidPrep Mini Spin Kit (GE Healthcare, Buckinghamshire, UK) as per manufacturer's recommendations.

For an easier identification of the clones, the following nomenclature was used: cat identification/sample source/presence (+) or absence (−) of FIP/cat origin/clone number.

### 2.5. DNA Sequencing

Sense primers were designed at approximately every 500 nucleotides to obtain full coverage of genes 3a–c, E, and M (Table 1). Primers for the plasmid (pUC/M13F and R) were also used to reach the full length of the amplicon sequence.

DNA sequencing with each primer in duplicate was performed using the BigDye*™* Terminator v3.1 Cycle Sequencing Kit (Life Technologies Corporation, Carlsbad, CA, USA) with a Genetic Analyzer 3500*™* capillary sequencer (Applied Biosystems Foster City, CA, USA), according to the manufacturers' instructions.

### 2.6. Sequence Editing

Chromatograms were generated in duplicate for each segment and evaluated for their base quality using the online application Phred (http://asparagin.cenargen.embrapa.br/phph/). Only those chromatograms with scores greater than 20 (probability lower than 1 error per 100 nucleotides) were used. The chromatograms were analyzed and manually edited with the software FinchTV version 1.4 (http://www.geospiza.com/Products/finchtv.shtml). The final sequences of each sample were obtained using the CAP contig application with the software BioEdit version 7.0.9.0 [22].

### 2.7. Phylogenetic Analyses

The nucleotides and amino acids corresponding to the 3a–c, E, and M genes were aligned using the application Clustal/W in BioEdit 7.0.9.0 [22]. Clones were classified in serotypes based on comparative analysis of the amino acid sequences encoded by the 3a gene with the sequences available in GenBank (serotype I: DQ160294 and DQ848678 and serotype II: DQ010921). Nucleotide phylogenetic trees (neighbor-joining, MCL model, 1,000 bootstrap replicates) were constructed using the computer software Mega version 5.05 [23].

## 3. Results

### 3.1. Messenger RNA Detection for the M Gene and Amplification, Sequencing, and Cloning of the 3a–c, E, and M Genes of FCoVs

mRNA was not obtained from all samples; nevertheless sequences for the 3a–c, E, and M amplicons were obtained from a total of 292 clones originating from 8 animals [6FIP+ (USP6, USP7, USP8, USP17, USP16, and USP18) and 2FIP− (USP2 and USP14)]. Sequences from extraintestinal tissue samples and faecal/intestines samples were obtained simultaneously for 3 animals (USP6, USP7, and USP17) with FIP (Table 2). Due to poor sequence quality for some clones and/or regions in a clone, not all full length amplicons could be sequenced or used in the analyses. The sequences obtained in the present study were deposited in GenBank under access numbers KJ879334 to KJ879438.

### 3.2. Phylogenetic Analysis of the 3a–c, E, and M Genes of FCoVs

**Figure 1:**
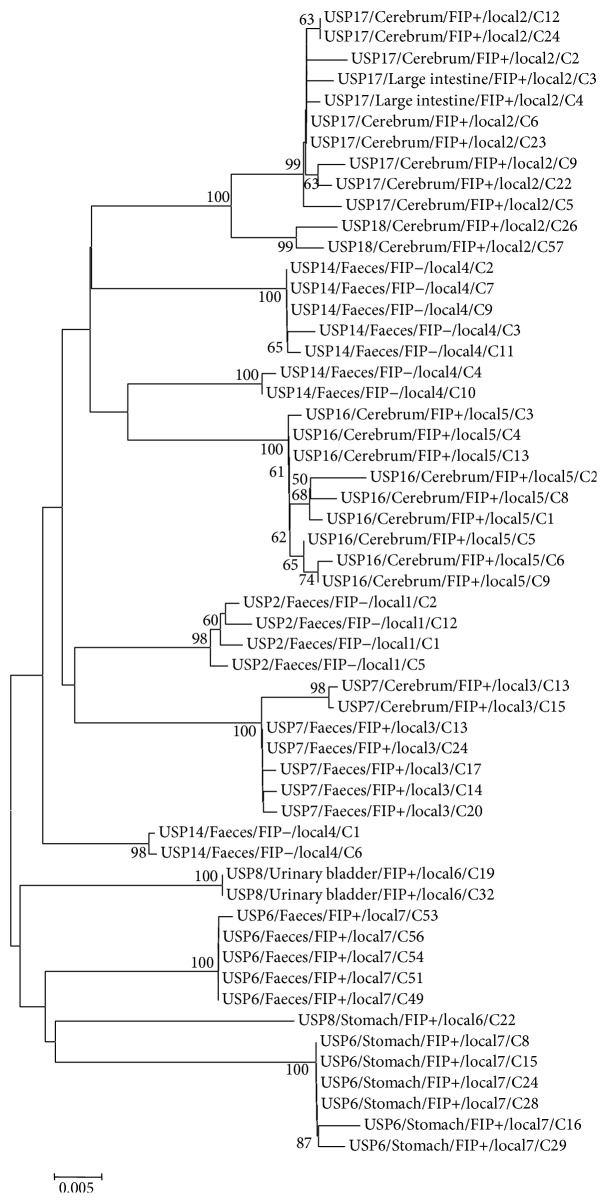
Phylogenetic tree constructed using the neighbor-joining method and the maximum composite likelihood substitution model for the FCoV 3c gene. The numbers next to each node represent the values of 1,000 bootstrap repetitions, and only those above 50% are shown. The scale represents the number of substitutions per site. For easy identification of the samples, the following nomenclature was used: cat identification/sample source/presence (+) or absence (−) of FIP/cat origin/clone number.

**Figure 2:**
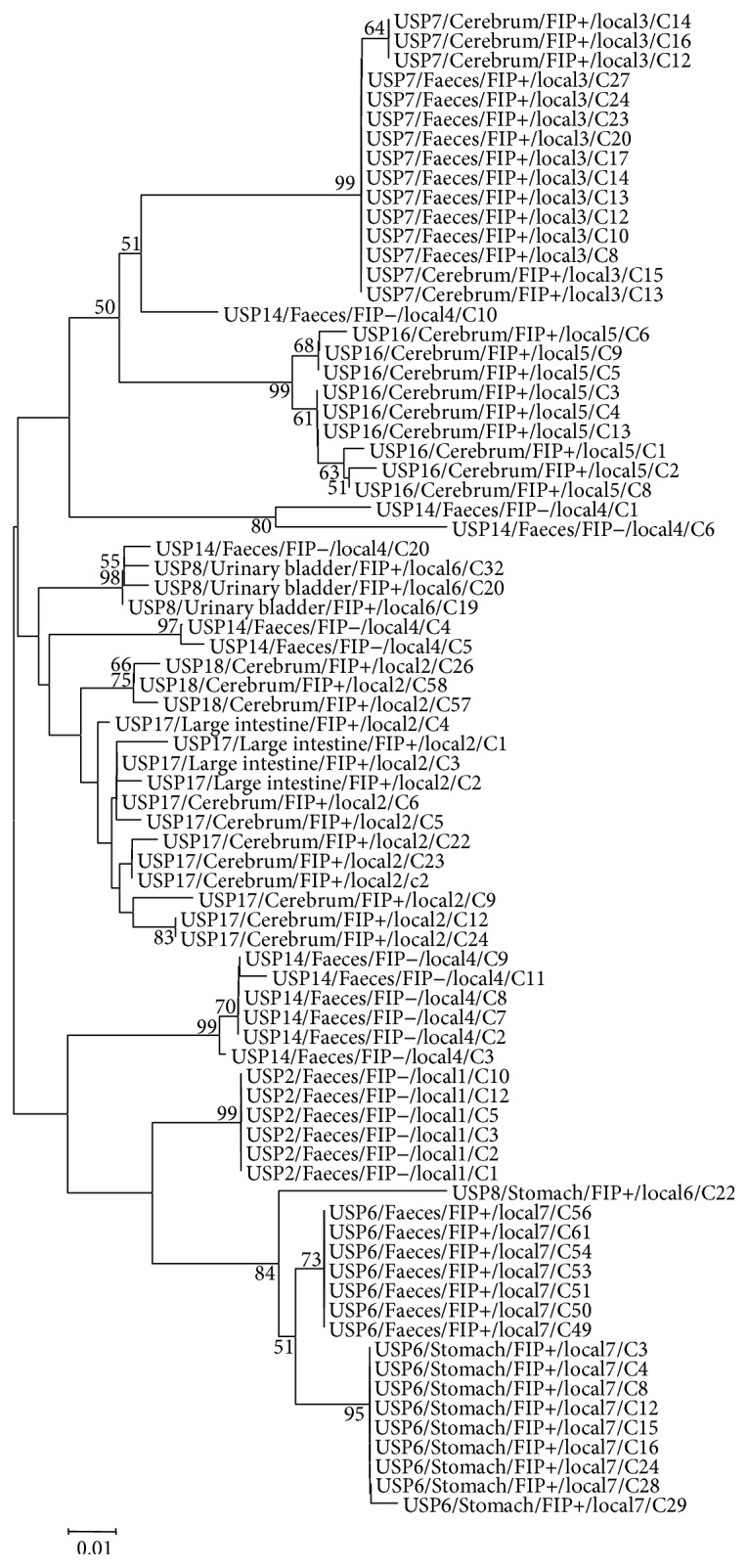
Phylogenetic tree constructed using the neighbor-joining method and the maximum composite likelihood substitution model for the FCoV 3b gene. The numbers next to each node represent the values of 1,000 bootstrap repetitions, and only those above 50% are shown. The scale represents the number of substitutions per site. For easy identification of the samples, the following nomenclature was used: cat identification/sample source/presence (+) or absence (−) of FIP/cat origin/clone number.

All sequences of the 3a genes obtained from a total of 79 clones belong to serotype I. Phylogenetic trees generated for the 3a, 3c, E, and M genes showed similar topologies and thus only the 3c and 3b trees are shown (Figures 1 and 2, resp.). Sample from cat USP14 (non-FIP) showed a remarkable clonal diversity as their clones are present in five different clusters of the 3b tree. High clonal diversity was observed in samples from FIP+ (USP8, USP16, USP17, and USP18) and FIP− cat (USP14); however FIP+ cats USP6 and USP7 and USP2 FIP− cat showed a lower clonal diversity. In the 3c gene tree, for cats USP6 and USP7, differences can be noticed between the FCoV strains obtained from tissue and faecal clones; that is, for each animal, there was a distinction between systemic (FIPV) and enteric (FECV) strains. However, no generic grouping per pathotype (FECV or FIPV) was observed for all samples and trees generated from this study.

Conversely, in the 3b gene tree samples obtained from cat USP7 (FIP+) could not be separated into systemic (cerebrum) and enteric (faeces) strains.

The overall nucleotide identity of all clones (FIP+ and FIP−) obtained from the 5 genes was ≥92.09% for faecal samples and ≥90.09% for tissue samples (Table 3). The greatest nucleotide diversity when all available sequences for each gene were compared together was observed for the 3b gene, for which a 90.09% identity was found, with the exception of comparisons between cats living in the same house, for which the identity between the 3b gene sequences was 97.09%.

For tissue and faecal samples, 3a was the most conserved gene (nucleotide identities of 99.38% and 97.70%, resp.) considering each individual cat. The mean identity between clones from the two cohabitating FIP+ cats (USP17 and USP18) ranged from 96.17% (3c gene) to 99.83% (E gene).

Premature stop codons were observed in the 3a (USP18, 3/3 cerebrum clones), 3b (USP17, 1/8 cerebrum; USP7, 1/10 faeces), 3c (USP17, 8/8 cerebrum and 2/2 large intestine; USP18, 2/2 cerebrum; USP7, 2/2 cerebrum; USP16, 1/9 cerebrum; USP8, 1/1 stomach; USP6, 6/6 stomach), and M (USP7, 1/5 cerebrum) genes from FIP+ cats. No premature stop codon in the 3a–c or E genes was observed in faecal samples from cats without FIP, with exception of M gene (USP2, 1/6 faeces).

Stop codons in 3c were evident in at least one of the clones from tissue samples from cats with FIP. The faecal samples from cats with or without FIP did not demonstrate this truncated gene in any of the clones, and no other molecular marker capable of differentiating the biotypes (FECV and FIPV) was observed in the sequenced genes.

## 4. Discussion and Conclusions

The existence of genetic markers to differentiate these two biotypes is currently a topic of the highest interest on the FIP field not only from the clinical and epidemiological points of view but also as a basis for an in-deep comprehension of the pathogenesis of this highly complex disease. Considering that, in the present study, clones of structural (E and M) and accessory (3a–c) genes were obtained from cats with and without FIP, and the results demonstrate a highly likely candidate FCoV pathotypic marker in the 3c gene.

Amplicons obtained from the cerebrum and large intestine of the cat USP17 (FIP+) were also cloned and sequenced. When assessing the 46 clones (32 clones from cerebrum and 14 from large intestine) obtained from this cat, the nucleotide identity among the five genes was greater than 99%, and according to the phylogenetic trees generated, clones from this cat resulted in a single cluster. In the 3c gene, a single nucleotide transversion (G → T) was observed at position 10 in all clones from samples from this cat, resulting in premature stop codons and truncated proteins. The observation of truncated proteins in large intestine observed herein is in contrast to what has been observed for FECV in other studies [13, 14, 20].

Possibly because it serves as a preferential organ for viral replication and therefore for pathological changes in FIP cases, the intestine of the cat USP17 (FIP+) was also affected by the systemic strain. Chang et al. [20] suggested that viruses with an inactivated 3c gene rarely replicate in the intestine, which helps to explain the rare incidence of FIP outbreaks due to a less frequent faecal-oral transmission. A premature stop codon in the 3c gene was previously reported in a strain of FCoV from the jejunum of a cat with FIP, and the same intestinal strain showed 100% identity with a strain obtained from a liver sample of the same cat [6].

In recent studies [13, 15], 3c genes with premature termination were detected in faecal and extraintestinal samples from cats with FIP, although it was not determined whether the viruses were replicating. Also, after an experimental inoculation of cats with truncated and complete 3abc, the role of the truncation in this region on FIP pathogenesis could not be clearly determined, despite it led to lower viral replication in the gut [24]. However, in the present study, the sample from the large intestine of the cat USP17 (FIP+), from which the studied clones originated and which demonstrated a truncated 3c gene, was positive for FCoV mRNA. This result indicated the presence of viral replication in that sample.

When comparing the sequences obtained from the extraintestinal organs and faeces of the FIP+ cats USP7 and USP6, the presence of a premature stop codon in the 3c gene was not observed in faecal clones from both cats, in contrast to what was observed for tissue clones. Moreover, the nucleotide identity of the 3c gene for tissue and faecal samples was 99.09% and 95.58% for the cats USP7 and USP6, respectively. In a study comparing FCoV from extraintestinal FIP lesions and the faeces of the same cat, the identity between the sequences of the structural and accessory genes evaluated (S, M, N, E, 3a–c and 7a-b) was always higher than 99%, corroborating the hypothesis of* in vivo* mutation [14].

As FIPV lineages may be derived from FECV mutations, it was expected that nucleotide diversity between faecal and tissue samples from the cat USP6 (FIP+) would be lower. However, the difference was 8.84% for the 3b genes, consisting exclusively in point mutations, indicating that this cat could be coinfected with an FCoV strain that is distinct from the systemic strain. Furthermore, the high nucleotide identity between samples from the cerebrum and faeces of the cat USP7 (FIP+), along with the topology of the generated trees, showed that the intestinal viral replication of the enteric pathotype may be maintained even during FIP in some cases, as demonstrated by the presence of mRNA.

As previously described and according with results from the present study, FIPV can both emerge endogenously and be transmitted to different cats as a “ready” virulent pathotype, which has major implications for the understanding of the dynamics of viral transmission [5, 15, 21].

Among the structural proteins genes (E and M) studied herein, premature stop codons were detected only in the M gene. The M protein of FCoVs is the most abundant structural protein [25]. The presence of a premature stop codon in a constitutive gene such as the M gene is incompatible with the functionality of the virion. However, this defective virus is no less important than the others, as it provides genetic background for recombination with other FCoVs, resulting in greater genetic diversity and possibly increased virulence of some viral quasispecies. Recent studies suggest that the 3c gene, which encodes a small protein (238aa) of unknown function, is associated with most FIPV isolates when present in truncated form, although not all FCoV strains in FIP+ samples present this 3c form [13, 14, 20]. In the present study, the 3c truncated protein was observed in all clones obtained from tissue samples from FIP+ cats, with the exception of those obtained from the cat USP16, where only 11% (1/9) of the clones demonstrated the truncated protein.

It is essential to note that if the predominant viral population in the present study had been assessed by direct amplicon sequencing only, as in studies conducted by other authors [13–15, 19, 20], without using the previous cloning step, the truncated 3c gene could have been undetected as it was only present in a minority of the sequences observed in the cerebrum of the cat USP16 (FIP+).

The transversions observed at position 10 of the 3c gene, which resulted in premature stop codons, were the same as those observed in the clone sequences obtained from the cerebrum and stomach of the FIP+ cats USP17 and USP6, respectively. With the exception of this finding and in agreement with the results of Pedersen et al. [14, 15], the 3c gene mutations were unique to each cat, including the sequences obtained from the cohabitating FIP+ cats (USP17 and USP18).

It can be concluded that the presence of a truncated 3c protein is a suggestive factor for differentiation between the FECV and FIPV pathotypes and therefore serves as a genetic marker of virulence. It still remains to be answered whether 3c is expendable for FCoV replication in phagocytic cells and a nontruncated 3c is thus selected-against during the FECV/FIPV transition. Moreover, the 3a–c, E, and M genes showed a level of genetic diversity indicative of the constitution of FCoV quasispecies or mutant spectrum and the likelihood of emergence of a highly virulent, individual-specific biotype.

## Figures and Tables

**Table 1:** Primers used to obtain full coverage of genes 3a–c, E, and M.

Primers	Sequence (5′-3′)	Position^a^
2B	YATYACWGTKTAYRABTT	487–505
3B	ACRACWYTNAKKTTTGTA	1058–1075
4B	AATAGCATTGCTAAATRT	1479–1496
5B	CGGCTTTAGTRTYGCAGG	1999–2008

^a^Position defined according to genome FCoV-WSU 79-1146 (DQ010921).

**Table 2:** Identification, sorting according to the presence of FIP detection of mRNA FCoVs, and the number of clones obtained from cats.

ID	Location	FIP	Sample	mRNA	Number of clones obtained from each gene					Total
					E	M	3a	3b	3c	
					249nt	792nt	213nt	222nt	714nt	
USP2	1	Absent	Faeces	Pos	4	6	6	6	4	26

USP6	7	Noneffusive	Stomach	Pos	10	11	10	9	6	46
			Faeces	Neg	0	3	8	7	5	23

USP7	3	Noneffusive	Cerebrum	Neg	5	5	5	5	2	22
			Faeces	Pos	3	7	10	10	5	35

USP8	6	Effusive	Stomach	Pos	1	1	1	1	1	5
			Urinary bladder	Pos	0	0	3	3	2	8

USP17	2	Effusive	Cerebrum	Neg	3	5	8	8	8	32
			Large intestine	Pos	3	1	4	4	2	14

USP14	4	Absent	Faeces	Pos	1	1	12	12	9	35

USP16	5	Effusive	Cerebrum	Pos	6	0	9	9	9	33

USP18	2	Effusive	Cerebrum	Pos	2	3	3	3	2	13

Total of clones (*n* = 292)					38	43	79	77	55	292

ID: identification; Pos: positive; Neg: negative.

**Table 3:** Mean, maximum, and minimum nucleotide identities of 3a–c, E, and M FCoV genes from different clones.

Identification	3a gene			3b gene			3c gene			E gene			M gene		
	Mean (%)	Max (%)	Min (%)	Mean (%)	Max (%)	Min (%)	Mean (%)	Max (%)	Min (%)	Mean (%)	Max (%)	Min (%)	Mean (%)	Max (%)	Min (%)
USP2/Faeces/PIF−/local1	99.83	100.00	99.50	100.00	100.00	100.00	99.45	99.50	99.40	99.75	100.00	99.50	99.52	99.80	99.30
USP6/Stomach/PIF+/local7	99.67	100.00	99.50	100.00	100.00	100.00	99.92	100.00	99.80	99.90	100.00	99.50	99.77	99.80	99.70
USP6/Faeces/PIF+/local7	100.00	100.00	100.00	99.03	99.50	98.60	99.20	99.20	99.20	*∗*	*∗*	*∗*	99.77	99.80	99.70
USP7/Cerebrum/PIF+/local3	99.02	100.00	98.10	96.39	100.00	88.10	99.79	100.00	99.70	99.53	100.00	99.10	99.48	100.00	99.40
USP7/Faeces/PIF+/local3	99.42	100.00	97.10	92.16	100.00	83.30	97.29	100.00	95.50	100.00	100.00	100.00	99.54	100.00	99.10
USP8/UB/PIF+/local6	98.70	99.50	98.10	99.89	100.00	99.50	99.73	100.00	99.20	*∗*	*∗*	*∗*	*∗*	*∗*	*∗*
USP14/Faeces/PIF−/local4	95.86	100.00	91.50	98.96	100.00	97.70	99.48	100.00	98.80	*∗*	*∗*	*∗*	*∗*	*∗*	*∗*
USP16/Cerebrum/PIF+/local5	99.42	100.00	99.00	99.33	99.50	99.00	100.00	100.00	100.00	99.83	100.00	99.50	99.70	100.00	99.40
USP17/Cerebrum/PIF+/local2	99.25	100.00	98.50	97.92	100.00	94.50	99.33	100.00	98.50	99.67	100.00	99.50	99.18	99.60	98.70
USP17/LI/PIF+/local2	99.50	100.00	99.00	99.03	99.50	98.60	99.50	99.50	99.50	99.67	100.00	99.50	*∗*	*∗*	*∗*
USP18/Cerebrum/PIF+/local2	98.70	99.50	98.10	98.68	100.00	98.10	99.80	99.80	99.80	100.00	100.00	100.00	99.53	99.60	99.40

Intestinal samples	97.70	100.00	91.50	92.09	100.00	81.50	95.64	100.00	93.20	93.87	100.00	91.10	99.59	100.00	99.10
Extraintestinal samples	99.38	100.00	98.10	90.09	100.00	75.60	95.37	100.00	92.70	95.19	100.00	89.90	99.53	100.00	98.70

Max: maximum; Min: minimum; LI: large intestine; UB: urinary bladder.

^*∗*^Samples that only one clone was obtained or no clone was obtained at all.
